# Surveillance of Obesity-Related Policies in Multiple Environments: The Missouri Obesity, Nutrition, and Activity Policy Database, 2007-2009

**Published:** 2010-06-15

**Authors:** Debra Haire-Joshu, Michael Elliott, Rebecca Schermbeck, Elsa Taricone, Scoie Green, Ross C. Brownson

**Affiliations:** Center for Obesity Prevention and Policy Research, George Warren Brown School of Social Work and School of Medicine, Washington University in St Louis; Washington University in St Louis, Saint Louis, Missouri; Washington University in St Louis, Saint Louis, Missouri; Washington University in St Louis, Saint Louis, Missouri; Washington University in St Louis, Saint Louis, Missouri; Washington University in St Louis, Saint Louis, Missouri

## Abstract

**Introduction:**

The objective of this study was to develop the Missouri Obesity, Nutrition, and Activity Policy Database, a geographically representative baseline of Missouri's existing obesity-related local policies on healthy eating and physical activity. The database is organized to reflect 7 local environments (government, community, health care, worksite, school, after school, and child care) and to describe the prevalence of obesity-related policies in these environments.

**Methods:**

We employed a stratified nested cluster design using key informant interviews and review of public records to sample 2,356 sites across the 7 target environments for the presence or absence of obesity-related policies.

**Results:**

The school environment had the most policies (88%), followed by after school (47%) and health care (32%). Community, government, and child care environments reported smaller proportions of obesity-related policies  but higher rates of funding for these policies. Worksite environments had low numbers of obesity-related policies and low funding levels (17% and 6%, respectively). Sixteen of the sampled counties had high obesity-related policy occurrence; 65 had moderate and 8 had low occurrences.

**Conclusion:**

Except in Missouri schools, the presence of obesity-related policies is limited. More obesity-related policies are needed so that people have access to environments that support the model behaviors necessary to halt the obesity epidemic. The Missouri Obesity, Nutrition, and Activity Policy Database provides a benchmark for evaluating progress toward the development of obesity-related policies across multiple environments in Missouri.

## Introduction

Policy initiatives are among the least understood but potentially most effective strategies for affecting the multiple environments contributing to the obesity epidemic (1). The prevalence of overweight and obesity has risen steadily in the United States by sex, age, race, and education for the past several decades (2,3). The rapid rise in obesity prevalence among young people and adults is attributable to multiple factors influenced by the environments in which people spend time, including community, worksite, and school (2,3). These environments frequently offer easy access to high-calorie foods and  limit physical activity because of automobile use and sedentary entertainment technologies (4). The rise of obesity across all populations and limited effects of individual interventions conducted in these environments led public health experts to call for policy-level changes designed to eliminate barriers to healthy choices (5,6).

Policies can be a strategy for making environmental changes because they encompass formal and informal rules, laws, and regulations (7,8). Several studies have assessed the effect of state and federal policies on food and activity behaviors in individual settings, including education, parks and recreation, and transportation (8,9). Despite early efforts under way to track state policy progress in chronic disease policy, local policy surveillance is lacking (10,11). The Environmental Nutrition and Activity Community Tool (ENACT) local policy database is an example of a voluntary repository for cataloging promising local policies related to obesity, which can inform local jurisdictions working to reshape schools, communities, and institutions (12). However, this repository does not reflect a representative sample of local policies gathered using random sampling from multiple environments. Instead, ENACT is an illustrative sample of well-created or particularly influential policies.

The goal of this study was to collect data to set a benchmark for the local obesity-related policy environment in Missouri (13,14). We had 2 objectives in accomplishing this goal. The first was to develop the Missouri Obesity, Nutrition, and Activity Policy (MoNAP) Database, a geographically representative baseline of Missouri's existing obesity-related local policies on healthy eating and physical activity, organized to reflect 7 local environments (government, community, health care, worksite, school, after school, and child care). The second was to describe the prevalence of obesity-related policies in these environments.

## Methods

We implemented a series of steps designed to identify obesity-related policies in Missouri. Our first step was to establish community partnerships so we could contact key informants from our 7 target environments. We collaborated with staff of the Prevention Institute, a leader in the development of the ENACT database, to learn from their methods and approaches (12). We also established an expert advisory group that reviewed MoNAP project goals, definitions, and data collection methods and provided initial contacts from each of our target environments. The advisory group consisted of educators, politicians, health care administrators, and members of state government recognized as leaders in obesity policy. Finally, we worked extensively with the Missouri Council of Activity and Nutrition (MoCAN), a coalition of representatives from groups interested in implementing the Missouri statewide obesity prevention plan. MoCAN has more than 46 active members from academia, business, health care, and other community groups. MoCAN members and workgroups were critical to educating the public about MoNAP and in securing extensive contact information in the target communities.

### Standardizing definitions

To ensure common language and consistency in assessing policies from each environment, we 1) identified a list of key terms, 2) conducted a literature search on commonly accepted definitions, 3) reviewed these definitions with key members of our team and advisory group, and 4) came to consensus about the meaning of our core constructs (12,15). We defined obesity-related policies as written documents describing a strategy, plan, or objective related to carrying out a physical activity or nutrition-related agenda (2). We used standard definitions and examples to guide the policy assessment of each of our target environments (Table 1).

### Study design and sampling plan

This study took place between 2007 and 2009. The Washington University in St. Louis institutional review board approved the conduct of this study. This study employed a stratified nested cluster design. The primary unit of sampling was the county; we obtained policies for sampled counties. The study team stratified the sample by using the 5 health regions as defined by the Missouri Department of Health and Senior Services. These regions are northwest (28 counties), central (29 counties), eastern (11 counties), southwest (24 counties), and southeast (22 counties).

We characterized each county as urban (>75% of residents living in an urbanized area or urban cluster), mixed residence (25% to 75% living in an urbanized area or urban cluster), or rural (<25% living in an urbanized area or urban cluster) and by racial/ethnic composition within county type. We drew the sample of counties proportionally to the number of counties in each health region and to the number of counties at each level of urbanization in each health region. For each county type (rural, mixed, and urban), we stratified by the relative racial/ethnic composition of the county (≥5% or more African American, 1% to <5% African American, and <1% African American). We oversampled counties with higher percentages of African Americans.

We next drew a stratified sample of cities from each of the sampled counties. We divided the cities within a county into tertiles based on population size. For each sampled city, we obtained policies from the 7 target environments. This approach enabled us to determine the presence or absence of obesity-related policies in 2,356 environments in 89 Missouri counties.

The government environment included city governments and special districts. The 114 counties in the state of Missouri necessitated a sample of 89 counties. (County-level policies were too broad to include.) The 972 cities in the state of Missouri required a sample of 276 city policies. We sampled city policies from each of the sampled counties (one from the highest, middle, and lowest tertile of city sizes), yielding a sample of 267 cities. Large cities were defined as those in the highest tertile (n = 89). Additionally, we forced the largest and smallest cities of each of the 5 regions into the sample for an additional 10 cities, yielding a total city count of 277.

For the community environment (eg, church associations or community centers) we sampled policies from 2 locations per city, yielding 554 locations for review*.* We identified 108 hospitals from the 23 health care systems throughout Missouri that provided coverage for the sampled cities. It was beyond the scope of this project to enumerate the number of worksites in the state of Missouri; however, we ensured representativeness by sampling policies from 2 worksites per sampled city (1 public worksite such as a park district office and 1 private worksite) for a total of 554 contacts for review. We defined school environment as the 432 school districts in the state of Missouri, requiring a sample of 204 districts. Because several cities are served by the same school district, a final target sample size of 217 was reached. Because of overlap in the school environments, we obtained 1 private after-school program policy (eg, YMCA) from each city. Finally, we sampled only child care centers licensed by the state (not family homes or group homes). One child care center was sampled from each sampled city, and an additional center was drawn from each large city (n = 92), yielding a total of 369 locations for review.

### Outcome measures

We used the MoNAP Policy Checklist (MPC) to assess the content of collected obesity-related policies. The MPC was based on the ENACT checklist and was modified to have 4 sections: demographics, topics, status, and funding. Demographic data included general information about the organization and key informants (eg, key informant name and title, organization name, policy name, city and county of organization site). The topics section included policy focus (eg, physical activity, nutrition, or both) and the presence or absence of obesity-related content (eg, access to fresh foods, body mass index reporting, land use/planning/zoning, rails to trails). The status section collected information on the type of policy (eg, city plan, ordinance) and addressed whether the policy was proposed or adopted. The funding section assessed whether the policy allocated funds for implementation. Informants were specifically asked whether funding was available and the source of that funding. Finally, 4 open-ended questions addressed history of policy development, policy adoption and implementation challenges, policy enforcement, and methods of policy evaluation by the organization.

### Data collection and analysis

We used 2 primary methods of data collection: key informant interviews and review of public documents. Key informant interviews were designed to generate a representative sample of policies and names of additional key informants from whom to gather policies. To identify these key informants, we worked with our advisory group and MoCAN to secure lists of contacts associated with each environment. For example, in the school environment, we identified people holding 1 of 4 positions: principals, physical education teachers, school nurses, and food service workers. We sent e-mails to these key informants explaining the purpose of the project and requesting samples of obesity-related policies. We followed the e-mails with a telephone call from project staff to review the project goals and ask key informants whether their organization had any written obesity-related policies and whether they would provide a copy of any written policy and names of other contacts. We made 3,666 contacts: government (n = 518), community (n = 444), health care (n = 299), worksite (n = 571), schools (n = 761), after school (n = 457), and child care (n = 616).

We also systematically reviewed public documents associated with each of the target environments. We conducted a Web-based search using various search tools (eg, Lexis, Nexis, Google). We identified relevant Web sites for the target environment (eg, schools) and key links to public policy-related documents (eg, school board meeting minutes), and collected any obesity-related policies or supporting documents. For example, for the government environment, we conducted Web-based searches to collect city council minutes, resolutions, and ordinances relevant to obesity-related policies.

We coded each site by information on policy status as *policy available*, *no policy available*, or *participation declined*. *No policy available* meant a verbal response of "no policy" or that we were unable to verify the presence of a policy. *Participation declined* meant a verbal response of "will not participate" or inability to contact the participant after at least 5 attempts (via telephone, e-mail, or both). Two project team policy analysts evaluated policies from "policy available" sites using the MPC. If there was disagreement in any MPC area, a third member of the project team assessed the policy and recommended category. There was 96% agreement among all policies collected. Most disagreements were due to omission as opposed to interpretation of content in each environment: government (24%), community (3%), health care (10%), worksite (2%), school (37%), after school (9%), and child care (15%). The difference in the rate of agreement for each environment was probably an artifact caused by capturing more policies in some environments than in others (eg, schools vs community). We used descriptive statistics to determine policy presence across each of the 7 environments.

## Results

The school environment had the highest rate of policies (88%), followed by after school programs (47%) and health care (32%) (Table 2). These environments reported that between 6% and 9% of their policies were funded. In contrast, community, government, and child care environments reported lower rates of obesity-related policies (5%, 17%, and 21%, respectively) but higher rates of funding for these policies (39%, 38%, 49%, respectively). Only the worksite environment reported low numbers of obesity-related policies and low funding levels (17% and 6%, respectively).

Counties with fewer than 2 environments covered by obesity-related policies were coded as low policy occurrence; 3 to 5 environments were coded as moderate policy occurrence; and 6 to 7 environments were coded as high policy occurrence (Figure). Only 16 of the sampled counties had high policy occurrence, most of which were in more highly populated areas of the state. In contrast, 8 counties had low occurrence, all counties were in more rural regions of the state. Most counties (n = 65) demonstrated moderate occurrence.

**Figure.**
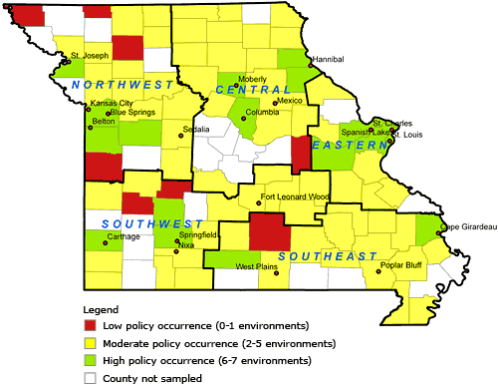
Environments with obesity policies, Missouri counties by region, Missouri Obesity, Nutrition, and Activity Policy Database, 2007-2009.

**Table:** 

In the central region, the number of obesity-related policies in each county were 7 for Marion; 6 for Boone, Ralls, and Randolph; 5 for Adair, Audrain, Camden, Clark, Linn, Macon, Putnam, and Shelby; 4 for Callaway, Chariton, Lewis, Montgomery, Schuyler, and Sullivan; 3 for Howard, Knox, and Monroe; and 2 for Gasconade. The following counties were not sampled: Cole, Cooper, Miller, Moniteau, Morgan, Osage, and Scotland.
In the eastern region, the number of obesity-related policies in each county were 7 for St. Louis; 6 for Franklin and St. Charles; 5 for Jefferson, and Washington; and 4 for Pike, St. Francois, Ste. Genevieve, and Warren. The following counties were not sampled: Lincoln, Perry, and Saint Louis City.
In the northwest region, the number of obesity-related policies in each county were 7 for Jackson; 6 for Buchanan, Cass, and Johnson; 5 for Caldwell, Carroll, Lafayette, Pettis, and Ray; 4 for Andrew, Benton, Clay, Grundy, Harrison, Mercer, and Saline; 3 for Clinton and DeKalb; 2 for Daviess and Worth, and 1 for Atchison and Bates. The following counties were not sampled: Gentry, Henry, Holt, Livingston, Nodaway, and Platte.
In the southeast region, the number of obesity-related policies in each county were 7 for Cape Girardeau; 6 for Douglas; 5 for Bollinger and Stoddard; 4 for Carter, Dunklin, Howell, Madison, Pemiscot, Reynolds, Scott, Shannon, Wayne, and Wright; 3 for Butler and Iron; and 2 for Texas. The following counties were not sampled: Mississippi, New Madrid, Oregon, Ozark, and Ripley.
In the southwest region, the number of obesity-related policies in each county were 7 for Greene and Polk; 6 for Jasper; 5 for Christian, Crawford, McDonald, Phelps, Pulaski, Stone, and Taney; 4 for Dent, Laclede, Maries, Newton, St. Clair, and Webster; 3 for Vernon, and 2 for Cedar and Hickory. The following counties were not sampled: Barry, Barton, Dade, Dallas, and Lawrence.

## Discussion

MoNAP provides a benchmark for assessing the presence of obesity-related policies across multiple environments in Missouri. Six of the 7 environments assessed reported a moderate occurrence of obesity-related policies. These data will allow for targeting of resources to improve the policy environment of areas with limited resources. From a research perspective, these data will provide a basis for understanding indicators of why policies are more likely to be addressed in 1 county rather than others (2,16).

MoNAP also revealed that schools were more likely to have a written obesity-related policy than any of the other 6 environments. This discrepancy with other environments might be best explained by the federal mandate in the Child Nutrition and WIC (Special Supplemental Nutrition Program for Women, Infants, and Children) Reauthorization Act of 2004, which required schools that sponsor school meal programs to establish wellness policies (2,17,18). These policies are a first step in ensuring that children spend their time in and have access to environments supportive of healthy eating and activity behaviors. In contrast, fewer than half of all after-school programs and only 1 in 5 child-care programs had obesity-related policies in place. This means that Missouri school children are more likely than children in after-school programs to have access to nutritious foods and activity for part of their day. This can be especially detrimental to young children, who are establishing lifelong patterns that may lead to obesity (15,19,20).

Only 32% of health care environments and 17% of government environments had obesity-related policies in place. Both of these settings, by virtue of their purpose and service, should be models for policies that promote public health through positive eating and activity (eg, tobacco control policies) (4,5). The same is true of worksite and community environments, settings where adults spend most of their day and where few obesity-related policies were in place (17% and 5%, respectively). However, the minimal presence of obesity-related policies offers little evidence that model environments exist. It also suggests that the current obesity epidemic, which contributes to rising health care costs related to chronic disease, is not yet recognized as a priority for most of these settings in Missouri.

Finally, this study provides directions for future work. Most obesity-related policies were unfunded (51%-94%). MoNAP did not assess the quality of the policies or the extent to which policies were implemented as designed. Work is under way to evaluate policy content, implementation, and its effect on obesity and related behaviors. Future research will also allow us to better understand the role of funding in policy implementation. MoNAP will provide the basis for future work that will assess the effect of the policy environment on Missouri obesity rates over time.

### Limitations

This study has limited generalizability because it was conducted in Missouri. Although we made every effort to discern the presence of a policy in each of the sampled sites, we were hampered by a lack of contacts, especially in smaller cities. Therefore, our data may do a better job of portraying the policy environment for medium-sized and large cities than for smaller cities. Additionally, our study relied on self-reported data, especially when the final disposition was "no policy." It is possible that a policy was in place but that the person we contacted was unaware of this. Finally, although the selection of the counties and the cities in the counties was random, the selection of sites within a city was not.

### Implications for practice

Healthy eating and physical activity are influenced by the multiple environments where people spend time (1,7,8,21). Obesity-related policies influencing these environments may create optimal conditions for positive behavior change and maintenance (4,5). This study provides a basis for examining the cumulative influence of the presence of obesity-related policies across multiple environments (eg, community, health care) on prevalence of obesity. MoNAP provides an objective benchmark regarding the presence of obesity-related policies across multiple environments in Missouri. Such an assessment is needed to enable practitioners and policy makers to determine how and where to intervene for the greatest effect. These data can also help state programs target areas in which policies need to be developed to promote healthy environments. Additional work will evaluate the quality of these policies, whether they are implemented as designed, and their effect on the obesity epidemic in Missouri (5,9).

### Conclusion

Obesity-related policies are a mechanism for ensuring population access to environments that support healthy eating and physical activity (22-24). MoNAP provides a mechanism for assessing the presence of obesity-related policies in Missouri. Our findings suggest that except for Missouri schools, the environments offer limited support for obesity-related policies. Substantial improvement is essential if the population is to have access to environments that support the model behaviors necessary to halt the obesity epidemic (1,19,25). MoNAP provides a benchmark for evaluating progress toward the development of obesity-related policies for multiple environments in Missouri.

## Figures and Tables

**Table 1:** Standard Definitions Used to Assess Environments — the Missouri Obesity, Nutrition, and Activity Policy Database, 2007-2009

Environment	**Standard Definition and Examples**
Government	Defined as city and as a state or local agency with political commitment, policy development, prioritized funding, and coordination of programs . . . to improve the health status of the population and reduce inequities in health status among population groups. Examples: city, city ordinances, transportation, parks and recreation, planning and zoning
Community	Defined as a group of people linked by geographic location to a nongovernmental entity. Examples: community civic centers, city, parks and recreation plan, land use plan, watershed plan, aging or senior centers, and YMCA
Health care	Defined as health care systems or organizations with "resources and activities . . . to influence health-related behavioral patterns and outcomes over time." Examples: breastfeeding and nutrition policies
Worksite	Defined as the location, public or private, of a person's occupation by which he or she earns a living and providing a controlled environment through existing channels of communication and social networks. Examples: employee wellness programs
School	Operationalized by district and defined as the physical location for reaching the nation's students in either a private or public setting during a typical 8-hour work day. Examples: student welfare and wellness program policies, school district wellness program
Afterschool	Defined as organized programs occurring during nonschool hours in both private and public settings. Examples: YMCA after-school programs, Boys and Girls Club programs
Childcare	Defined as a place maintained by any person who provides care for more than 4 children during the day, for compensation or otherwise, except those operated by a school system. Examples: Head Start, Missouri Area Agency on Aging policies

Abbreviation: YMCA, Young Men's Christian Association.Sources: references 12 and 15

**Table 2:** Summary of Obesity-Related Policies by Target Environment — the Missouri Obesity, Nutrition, and Activity Policy Database, 2007-2009

Target Environment, n	Policy Available		No. With No Policy Available **(%)** n = 1,528	No. That Declined Participation **(%)** n = 227

	No. With Policy Collected **(%)** ** N = 601 **	% Funded,^a^ **n = 101**		
Government, 277	47 (17)	38	208 (75)	22 (8)
Health care, 108	35 (32)	6	51 (47)	23 (21)
Community, 554	28 (5)	39	478 (86)	48 (9)
Worksite, 554	93 (17)	6	419 (76)	42 (7)
School, 217	192 (88)	8	9 (4)	16 (8)
Afterschool, 277	130 (47)	9	104 (38)	43 (15)
Childcare, 369	76 (21)	49	259 (70)	34 (9)

a Funded policies are defined as those that mandated change and included monies to support implementation of those changes; unfunded policies are defined as those that mandated changes but did not fund entities to implement those changes.
